# Editors of the Monthly to Its Subscribers

**Published:** 1860-03

**Authors:** 


EDITOR OF THE MONTHLY—The following resignation was placed in the hands of the Faculty of the New York Medical College more than two months ago. To prevent any misapprehension that may exist in regard to the step I have taken, I have to ask the favor of the publication of the accompanying letter of resignation in the MONTHLY. H. G.

*To the Faculty of the New York Medical College:*

GENTLEMEN—Having put into the hands of the Trustees my resignation of the Chair held by me in the New York Medical College, to take effect at the close of the present term of Lectures, I now resign into your hands the Presidency of the Faculty, also to take effect at the close of this Course.

This step, which I have long contemplated, I feel compelled to take, inasmuch as my laborious professional duties, the duty I owe to a large family, and particularly a due regard for my health, will not allow me to discharge my duties to the College, with satisfaction to myself, or benefit to the School. The present period, moreover, seems a fit time for taking this course. A most efficient and excelent corps of Professors the Trustees have now secured, and the indications of success, and of the future prosperous condition of the School, were never more promising than at the present time.

This step has been taken after mature deliberation, and with much regret at withdrawing from a School with which, from its origin, I have been connected, and over whose Faculty, through a period of ten years, I have had the honor of presiding. But, still more deeply do I regret a separation from colleagues with whom I have passed so many years of pleasant and agreeable intercourse in our mutual endeavors to advance Medical Science.

Wishing you, gentlemen, individually, happiness and prosperity, and earnestly hoping that increased and permanent success may attend the Institution with which you are connected,

I remain, respectfully, your ob't serv't, HORACE GREEN.

NEW YORK, *Jan. 2d*, 1860.

